# Supplementary material for: Genomic differentiation and intercontinental population structure of mosquito vectors Culex pipiens pipiens and Culex pipiens molestus
Source: Sci Rep. 2020 May 5;10:7504. doi: 10.1038/s41598-020-63305-z (PMC7200692; doi:10.1038/s41598-020-63305-z)
Supplement: Supplementary file 1 — Supplementary information. [file 41598_2020_63305_MOESM1_ESM.pdf]

Genomic differentiation and intercontinental population structure of mosquito vectors *Culex pipiens pipiens* and *Culex pipiens molestus*

Andrey A. Yurchenko, Reem A. Masri, Natalia V. Khrabrova, Anuarbek K. Sibataev, Megan L. Fritz, and Maria V. Sharakhova

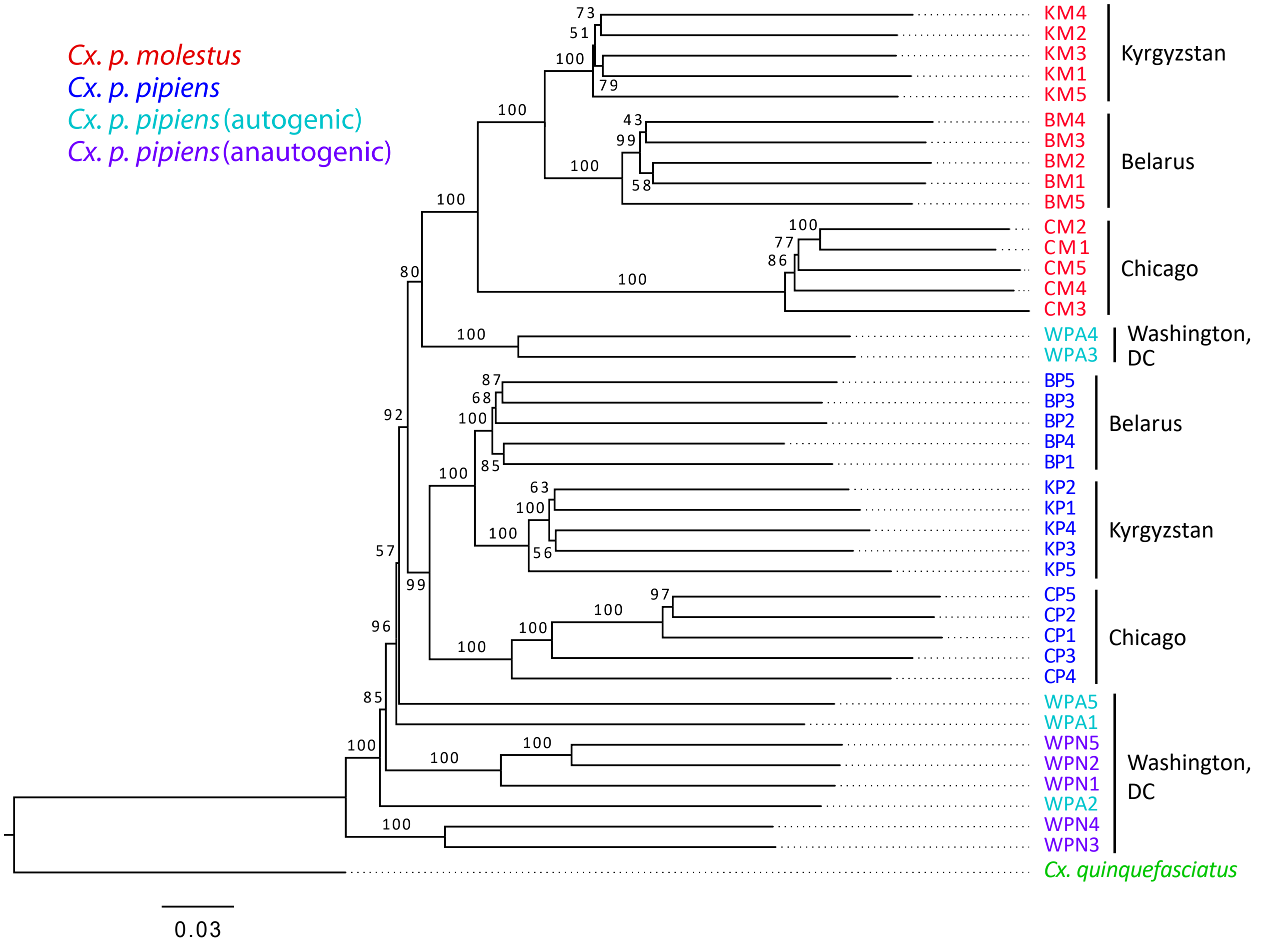

Supplementary Fig. 1. Neighbour-Joining tree constructed with nuclear dataset.

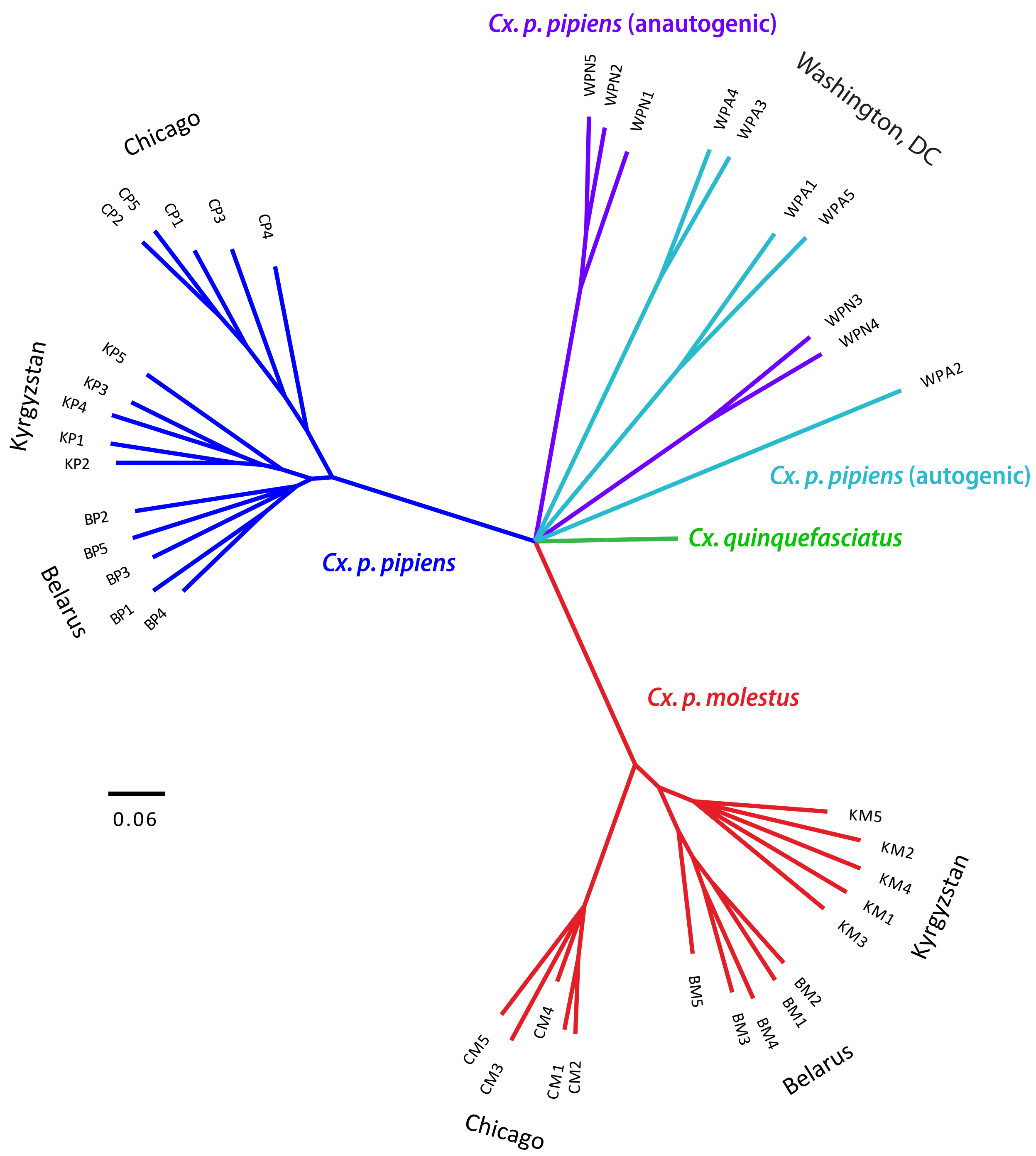

Supplementary Fig. 2. Maximum-Likelihood rooted tree constructed with nuclear dataset. All the nodes with bootstrap values lower 90% are collapsed.

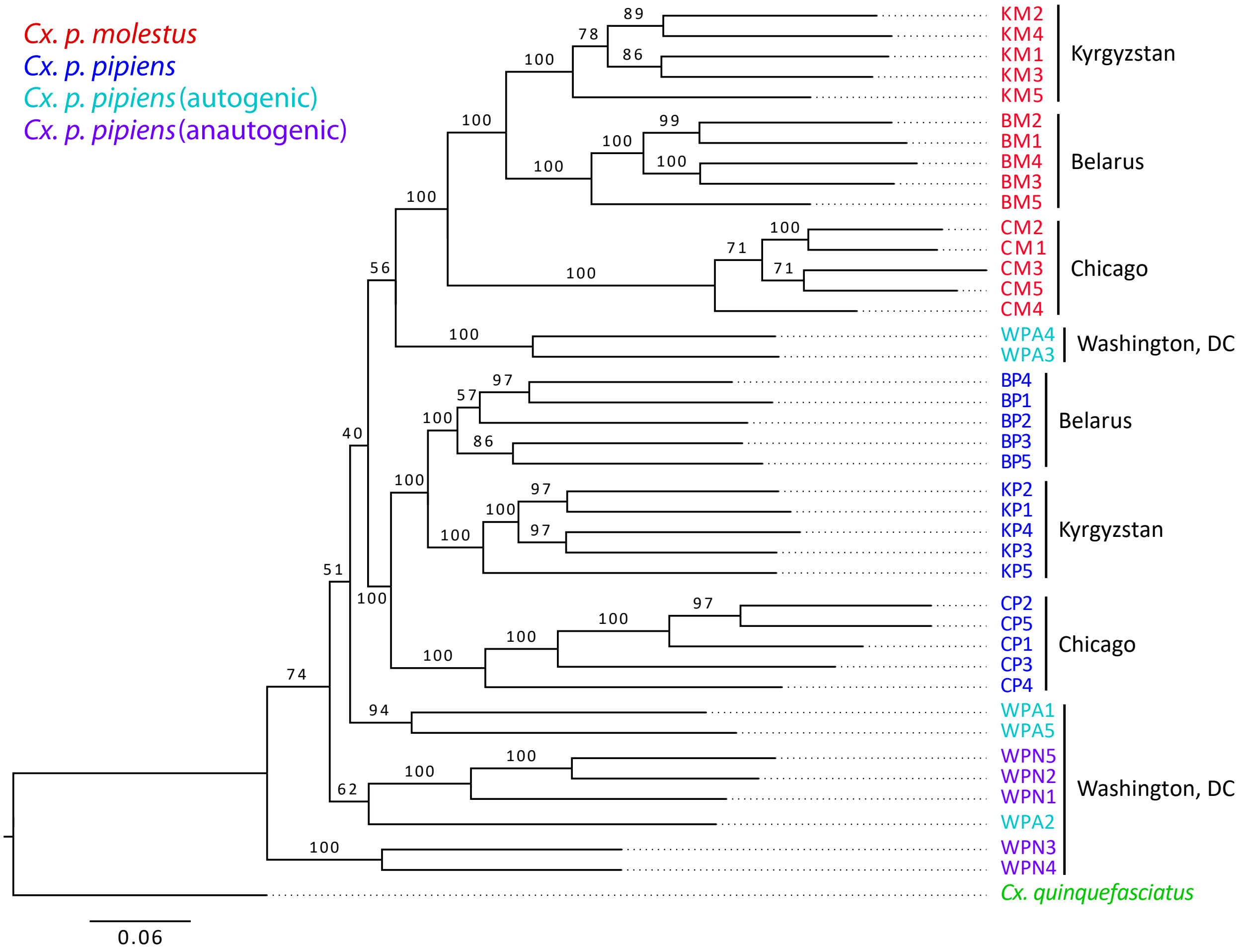

Supplementary Fig. 3. Maximum-Likelihood rooted tree constructed with nuclear dataset and rooted to *Cx. quinquefasciatus*.

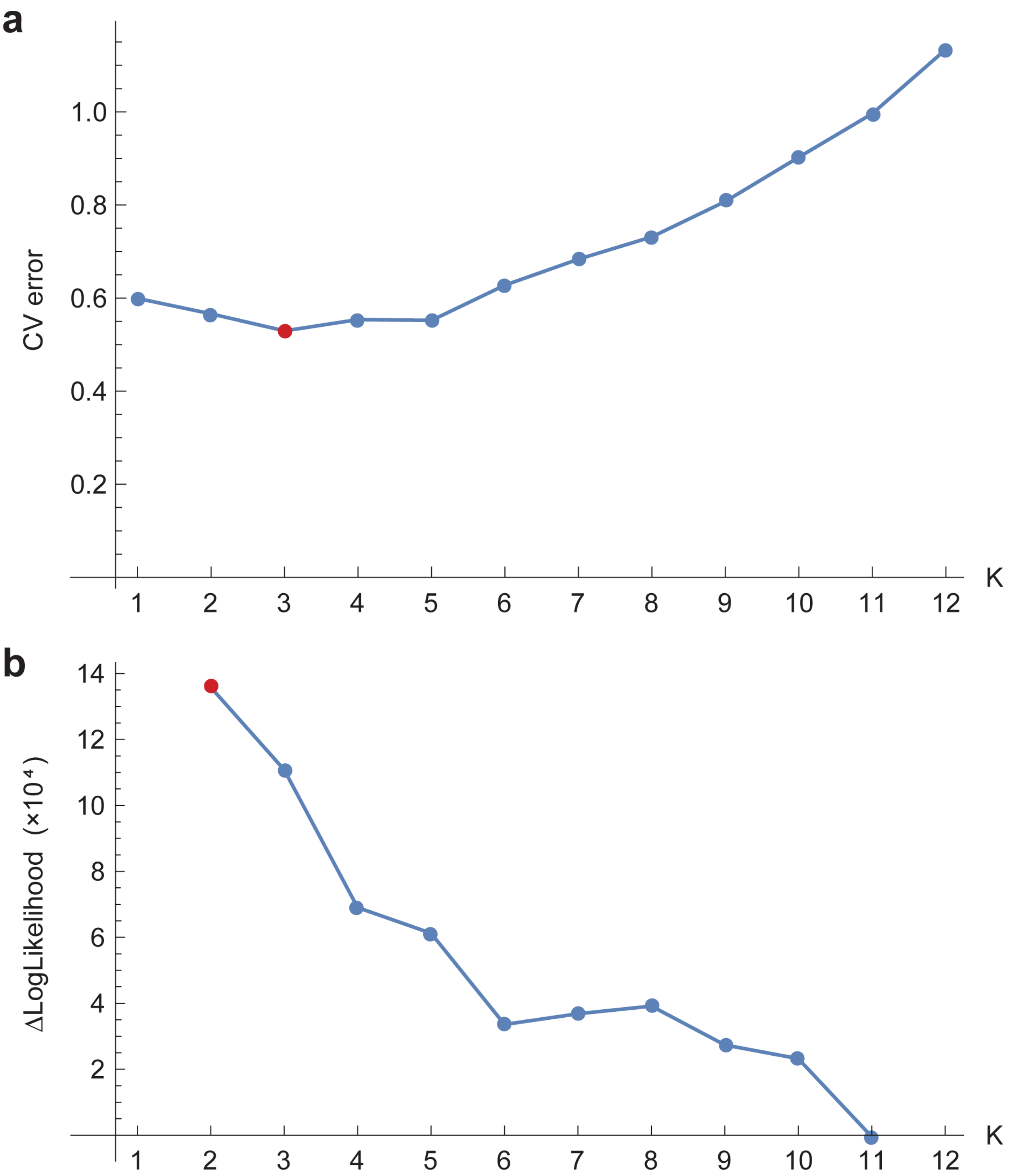

Supplementary Fig. 4. ADMIXTURE Cross-Validation error (A) and Δ Loglikelihood (B) point out to K=2-3 as the optimal number of genetic clusters.

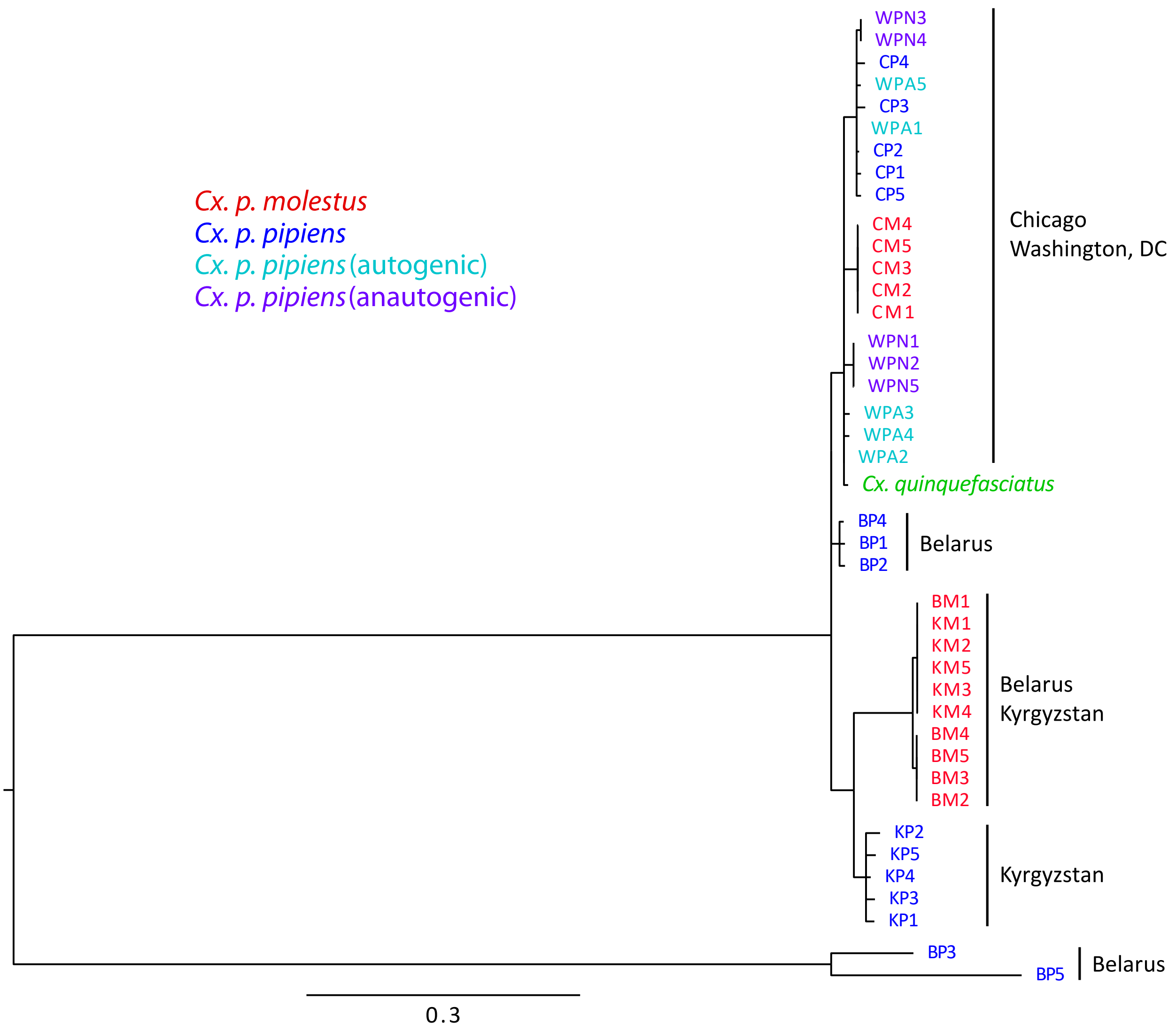

Supplementary Fig. 5. Maximum-Likelihood rooted tree constructed with mitochondrial dataset. All the nodes with bootstrap values lower 70% are collapsed.
